# Anthropometric and sociodemographic variables, but not preconception or prenatal maternal nutrition supplementation, predict neurodevelopment in offspring of the ‘Women First’ trial

**DOI:** 10.1111/mcn.13703

**Published:** 2024-07-23

**Authors:** Stephanie Waldrop, Dhuly Chowdhury, Jamie E. Westcott, Fred Biasini, Ana Garcés, Lester Figueroa, Antoinette Tshefu, Adrien Lokangaka, Melissa Bauserman, Sarah Saleem, Sumera A. Ali, Robert L. Goldenberg, Shivaprasad S. Goudar, Sangappa M. Dhaded, Richard J. Derman, Jennifer F. Kemp, Marion Koso‐Thomas, Abhik Das, Michael Hambidge, Nancy F. Krebs, The Women First Preconception Nutrition Trial Study Group

**Affiliations:** ^1^ Department of Pediatrics, Section of Nutrition University of Colorado School of Medicine Aurora Colorado USA; ^2^ RTI International Durham North Carolina USA; ^3^ Department of Psychology University of Alabama at Birmingham Birmingham Alabama USA; ^4^ Instituto de Nutrición de Centro América y Panamá (INCAP) Guatemala City Guatemala; ^5^ Kinshasa School of Public Health (KSPH) Kinshasa Democratic Republic of the Congo (DRC); ^6^ Neonatal‐Perinatal Medicine University of North Carolina Chapel Hill North Carolina USA; ^7^ Department of Community Health Sciences Aga Khan University Karachi Pakistan; ^8^ Department of Obstetrics and Gynecology Columbia University New York New York State USA; ^9^ KLE Academy of Higher Education and Research (Deemed‐to‐be‐University), Jawaharlal Nehru Medical College (JNMC) Belagavi India; ^10^ Office of Global Affairs Thomas Jefferson University Philadelphia Pennsylvania USA; ^11^ Eunice Kennedy Shriver National Institute of Child Health and Human Development Bethesda Maryland USA

**Keywords:** infant neurodevelopment, linear growth, maternal nutrition supplement, nurturing care, preconception, SQ‐LNS

## Abstract

Multiple factors influence infant and child neurodevelopment in low resource settings. In offspring of participants in the preconception maternal nutrition trial, Women First (WF), we examined the impact of providing a preconception (Arm 1) or prenatal (Arm 2) nutrient supplement (compared to controls, Arm 3) on neurodevelopmental outcomes at 24 months; predictors of neurodevelopment scores; and associations of infant anthropometrics with neurodevelopmental scores. Follow‐up visits for anthropometry were conducted at 6‐, 12‐, 18‐ and 24‐month of age. At 24‐months, in a randomized subset, the Bayley Scales of Infant Development, 3rd edition (BSID‐III), including cognitive, motor and social‐emotional subscales, and the Family Care Indicators (FCI) questionnaire, assessing family and home environment, were completed. Multiple covariates (intervention arm, site, maternal sociodemographic characteristics, FCI subscales, birthweight and 6–24 months' change in anthropometry *z*‐scores, (e.g., ΔLAZ_6–2__4_) were evaluated by linear regression to predict BSID‐III outcomes and to assess associations of anthropometric changes with BSID‐III scores. The analysis consisted of 1386 infants (*n* = 441, 486, 459 for Arms 1, 2 and 3, respectively). None of the domain‐specific BSID‐III subscale scores differed by maternal intervention arm. Four covariates significantly predicted (*p* ≤ 0.01) all 3 BSID‐III subscales: secondary maternal education, ΔLAZ_6__–__24_, birthweight >2500 g, and FCI play materials. Linear growth was associated with all domains of neurodevelopment. The results underscore the multi‐dimensional aspects of child development represented by the nurturing care framework, including prenatal maternal nutrition, post‐natal growth, maternal education for responsive caregiving and opportunities for early learning.

## Key messages

- Preconception and prenatal maternal nutrition is important to ensure adequate fetal growth and healthy birthweight.
- Preconception and prenatal maternal nutritional supplementation was not associated with young child neurodevelopment but has been shown to improve post‐natal growth.
- Post‐natal linear growth is consistently associated with offspring neurodevelopment at 24 months.
- Maternal education to foster responsive caregiving, and opportunities for early learning in the home also positively impact offspring neurodevelopmental outcomes.
- These findings underscore the importance of multi‐dimensional aspects essential for children to reach their full developmental potential.

## Graphical abstract

The study examined the effects of maternal nutrition supplementation started before or during pregnancy (stopped at delivery) on offspring development at 24 months of age. The maternal intervention was not significantly associated with neurodevelopment, but maternal education, fetal and post‐natal growth and home environment were all associated with better developmental outcomes.

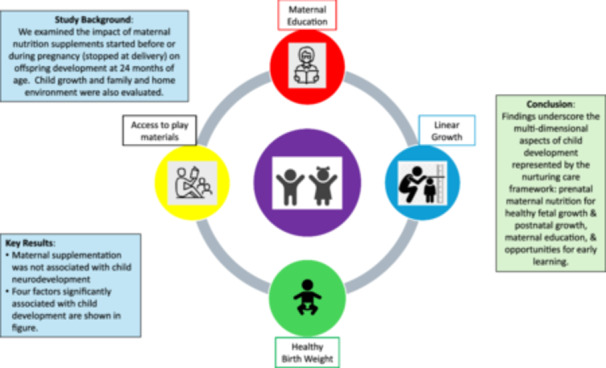

## 1 INTRODUCTION

Maternal preconception and prenatal nutrition are critical environmental influences that are potentially modifiable and may alter the foundations of infant brain development (Cusick & Georgieff, 2016). The neurodevelopmental vulnerability of the fetus and the phenotypic profile that result from any specific nutrient deficit are dependent on the timing of nutrient deficiencies and brain region‐specific nutrient requirements (Georgieff et al., 2018). However, developmental outcomes are also impacted by both the child's direct and reciprocal interaction with his or her environment (Prado & Dewey, 2014). Nurturing family and home environments have been shown to attenuate the effects of adverse early life experiences and improve long term cognitive outcomes (Trude et al., 2021).

While it is biologically plausible that maternal nutritional status in early gestation may contribute to offspring neurodevelopment, evidence of its direct influence across multiple neurodevelopmental domains is conflicting (Veena et al., 2016). One preconception trial of iron and folic acid supplementation found positive impact on one component of motor development (Nguyen et al., 2017). In contrast, another found impact on cognitive and language development from vitamin B12 supplementation alone but not in combination with a multiple micronutrient supplement (D'Souza et al., 2021). Thus, in low‐and middle‐income countries (LMICs) with high prevalence of maternal undernutrition, nutritional supplementation may be beneficial to child neurodevelopmental outcomes (Stephenson et al., 2018), especially in the context of the myriad of environmental and social factors that also exert influence (Bhutta et al., 2017; Black et al., 2021; Prado, Abbeddou, Adu‐Afarwuah, et al., 2017). Improved maternal nutritional status, however, may be insufficient alone, and the provision of ‘nurturing care’, characterized as ‘…a home environment that is sensitive to children's health and nutritional needs, responsive, emotionally supportive, and developmentally stimulating and appropriate, with opportunities for play and exploration and protection from adversities’ is also required (Black et al., 2017, 2021; Trude et al., 2021; World Health Organization, United Nations Children's Fund, & World Bank Group, 2018). Consideration of such potentially synergistic influences has implications for the breadth and sustainability of interventions designed for LMIC.

The current report presents neurodevelopment outcomes in the offspring of participants in the Women First Preconception Maternal Nutrition trial (WF) trial (Hambidge et al., 2014). These outcomes are based on the Bayley Scales of Infant Development, 3rd edition (BSID‐III) (Bayley, 2006) administered at 24 months of age. In the WF study, a three‐arm individually randomized controlled trial conducted in low resources settings, a small‐quantity lipid based nutrient supplement (SQ‐LNS) initiated before conception or in early gestation and continued through pregnancy, improved fetal growth and birth anthropometry (length and weight) and post‐natal growth through age 6 months (Hambidge et al., 2019; Krebs et al., 2021). In turn, birth length was a strong predictor of length and risk of stunting at 24 months of age (Krebs et al., 2022).

Our objectives for the current study were to determine: (1) the impact of preconception or early pregnancy provision of the SQ‐LNS intervention (compared to controls) on offspring neurodevelopmental outcomes at 24 months; (2) anthropometric and sociodemographic predictors of these neurodevelopment scores; and (3) the relationship between changes in length, weight and head circumference from 6 to 24 months and neurodevelopmental scores. We hypothesized that the provision of a preconception SQ‐LNS (Arm 1) as compared to early prenatal SQ‐LNS (Arm 2) or no SQ‐LNS (Arm 3) would result in higher neurodevelopmental scores and that components of post‐natal growth would be positively associated with neurodevelopment outcomes.

## 2 METHODS

### 2.1 Study design

This analysis included neurodevelopmental testing at 24 months and prospectively obtained anthropometry on a randomly assigned subset of live‐born infants of women in the WF Trial (Hambidge et al., 2019).

The primary WF study was a multisite individually randomized clinical trial designed to test the effects on newborn size, especially length, of commencing nutrition supplements for women in low resource settings before conception compared with the same supplement commenced late in the first trimester of pregnancy. Nonpregnant women were randomized to 1 of 3 intervention arms to assess the effect of SQ‐LNS on offspring anthropometrics at birth. The SQ‐LNS (Nutriset) provided 21 micronutrients, modest quantities of protein and energy (2.6 g protein and 118 kcal) and polyunsaturated fatty acids (5.2 g) in a favourable balance (Hambidge et al., 2014). Women in Arm 1 (*n* = 748) were provided the SQ‐LNS at enrolment and for least 3 months before conception through pregnancy. Women in Arm 2 (*n* = 806) started the SQ‐LNS at 12 weeks of gestation through pregnancy, while women in Arm 3 (*n* = 770) served as a control arm, receiving the same study monitoring as participants in Arms 1 and 2 and standard prenatal care, but with no nutrition supplementation provided by the trial. When the SQ‐LNS was initiated, women in Arms 1 and 2 who were underweight or found to have inadequate gestational weight gain (~90% of participants) were also provided an extra balanced protein‐energy supplement (Nutriset) that provided up to 300 kcal and 11 g of protein (~15% of energy) without additional micronutrients (Hambidge et al., 2014). Study interventions were discontinued at delivery. The WF trial was conducted in low resource, rural, and small town settings of 4 LMIC: the Democratic Republic of the Congo (DRC) (Equateur, *n* = 1741), Guatemala (Chimaltenango, *n* = 1808), India (Belagavi, *n* = 1823) and Pakistan (Thatta, Sindh Province, *n* = 2015), all of which were participating sites of the *Eunice Kennedy Shriver* National Institute of Child Health and Human Development Global Network (GN) for Women's and Children's Health Research (Koso‐Thomas & McClure, 2015). Additional details regarding the original study design and outcomes of the primary trial have been previously described (Hambidge et al., 2014, 2019). Briefly, birth length and weight did not differ between Arms 1 and 2, but both were significantly greater than those for Arm 3. Post‐natal growth was monitored in the WF offspring at 6 month intervals through 24 months of age. Although maternal treatment arm was significantly associated with the trajectory of post‐natal linear growth, stunting rates at 24 months were greater than 60% for infants from all 3 intervention arms (Krebs et al., 2022).

### 2.2 Participants

The young children in the present study are a randomized subset of offspring of participants from the Women First trial, representing approximately two‐thirds of the infants with valid birth measurements and whose parents consented to having them be followed until 24 months. Anthropometric measurements were obtained at 6, 12, 18 and 24 months of age; and the neurodevelopmental assessments were performed at 24 months of age. The participating children (49% male, 88% term) were assessed for neurodevelopment using the BSID‐III as shown in the CONSORT diagram (Supporting Information S1: Figure S1). The other one‐third of WF offspring were evaluated according to the INTERGROWTH‐21st Neurodevelopment Assessment (INTER‐NDA) standards, which were specifically designed for use in low resource settings (Fernandes et al., 2020, 2023). The children included in the present analyses had at least one BSID‐III subscore assessed at age 24 months. Data were collected between February 2015 and May 2017. Participants with congenital anomalies known to be associated with neurologic deficits severe enough to preclude testing were excluded. Gestational age was not an inclusion criterion. The recruitment process and the inclusion and exclusion criteria are shown in Supporting Information S1: Figure S1. Lost to follow‐up was minimal with a total of 1386 participants (93%) considered for neurodevelopmental analysis and 1384 with complete anthropometric measures at from 6 to 24 months of age and with BSID scores at 24 months of age.

The central data coordinating centre (RTI International) created the initial randomization scheme, which included a permuted block design stratified by GN site with a trial arm allocation ratio of 1:1:1 within blocks to include approximately two‐thirds of infants, evenly distributed across arms and sites.

The sample size was determined to provide sufficient power to detect a statistically significant mean difference of 0.9 (or 1.1) or greater with 80% (or 90%) power (assuming a two‐sided test with overall 5% Type I error for individual site analyses, while correcting for multiple comparisons across the three study arms.

### 2.3 Anthropometry

Birth anthropometric measurements, including length, weight and head circumference, were obtained within 7 days of birth; 98.2% were obtained within 48 h of birth by trained site personnel. The follow‐up anthropometry at 6, 12, 18 and 24 months of age was obtained by assessors who were trained, certified and re‐certified approximately every 3 months. The procedures and equipment used and the use of the World Health Organization (WHO) Child Growth Standards for calculation of age‐ and sex‐based anthropometric *z*‐scores have been reported previously (Hambidge et al., 2014; Krebs et al., 2022).

### 2.4 Neurodevelopmental assessment

The BSID‐III was used to evaluate developmental outcomes in cognitive, motor, and social‐emotional subscales and the risk of cognitive or motor developmental delay (i.e., a composite cognitive score <75 or a composite motor score <75) at 24 months of age. The BSID‐III has previously been validated in low and middle income countries (Del Rosario et al., 2021), including extensively in the GN sites. Initial centralized training of neurodevelopment assessment specialists, led by the neurodevelopment content expert for the Global Network (F. B.), was conducted for the WF site neurodevelopment specialists before implementation of the on‐site testing. Cognitive and motor domains were assessed by the specialists on site, and the social‐emotional behaviours were determined by parental report. There were typically 2 BSID evaluators at each site; all were certified in BSID administration, and all were blinded to the original maternal randomization assignment. Parents were present but not allowed to assist their infants during the assessment. As necessary, modifications of the study test materials were performed to ensure appropriate comparable comprehension for study subjects at each site and to assure fidelity to the intent of the question (Wallander et al., 2010). Regular surveillance of test administration and scoring for each site was conducted throughout the study in person by the local neurodevelopment specialists and via videotaping by the GN neurodevelopment specialist (F. B.).

### 2.5 Child home environment assessment

The Family Care Indicators (FCI) questionnaire (Hamadani et al., 2010), a survey‐based indicator of home environment quality as pertains to child development, was administered to care providers of all participants at the time of the 24 month neurodevelopmental assessments. This questionnaire was originally developed and validated for LMIC settings to assess child stimulation within the home and is based on parental responses regarding five subscales, including play activities, varieties of play materials, sources of play materials, household books, and magazines and newspapers. (Hamadani et al., 2010).

### 2.6 Statistical analysis

All developmental scores and assessments for cognitive, motor and social‐emotional domains were included in the analyses, which were conducted across all sites. The plots of the residuals for BSID‐III cognitive, motor and social‐emotional score outcomes revealed each to be normally distributed. The use of a general linear model approach allowed the assessment of mean differences between respective continuous outcomes of treatment arms while adjusting for pertinent covariates (viz, intervention arm, site, maternal sociodemographic characteristics, FCI subscales, birthweight and 6–24 months' change in anthropometry *z*‐scores, [e.g., ΔLAZ_6_–_24_]). Binary outcomes (i.e., composite developmental cognitive or motor scores ≤75 or >75) were analyzed using logistic regression with adjustments made for the same aforementioned covariates. Interaction effects between specific subgroups (such as sites) and intervention arm were considered in the adjusted models to assess relevant subgroup effects. A priori‐determined covariates, such as multiple sociodemographic characteristics and FCI, allowed for the evaluation of the quality of a child's home environment on offspring neurodevelopmental outcomes. All models adjusted for intervention arm and randomization stratification factors (site and cluster). The specific study aims, regression models employed and adjusted covariates evaluated are detailed below.

Aim 1: To determine whether preconception or early pregnancy provision of the SQ‐LNS intervention influenced neurodevelopmental outcomes, we fit models as described above, examining the effect of the three treatment arms while adjusting for factors that were different across the three randomized groups in this follow‐up analysis cohort. For the continuous outcomes, BSID scores, all final models included the following covariates: treatment arm, site, cluster, interaction between site and cluster, maternal education, maternal age, parity, socioeconomic status (SES), infant sex and FCI subscales: play activities (subscale score 0–10) and play materials (subscale score 0–8). For the categorical outcomes of cognitive scores <75 and motor scores <75 indicating developmental delay, the final models included the following covariates: maternal education, maternal age, parity, infant sex and FCI subscales. With respect to the categorical analysis, model convergence issues due to low cell counts/frequency did not allow us to evaluate for interaction effects with site and cluster in the final model.

Aim 2: To determine predictors of neurodevelopment scores regardless of treatment arm, we adjusted for all factors identified in Aim 1, as well as low‐birthweight status, length‐for‐age *z*‐score, weight‐for‐age *z*‐score and head circumference‐for‐age *z*‐score (LAZ, WAZ and HCAZ, respectively) at 6 months of age. A priori‐determined covariates (see above), including sociodemographic characteristics and FCI, allowed for the evaluation of the quality of a child's home environment on offspring neurodevelopmental outcomes. Generalized linear models were used to assess adjusted mean differences in BSID‐III composite scores. Analyses of binary categorical composite developmental score outcomes were performed for cognitive and motor domains only, using logistic regression models to estimate likelihood ratios with a 95% confidence interval (95% CI).

Aim 3: To determine the relationship between linear growth and other anthropometric indicators from infancy to 24 months with neurodevelopmental scores, we fitted the same models as in Aim 2 (see above for covariates), also adjusting for change in LAZ, WAZ, and HCAZ from 6 months to 24 months of age; and LAZ <−2 (stunting); and WLZ <−2 (wasting). The WAZ score at 6 months was excluded from the final model as it proved highly correlated with LAZ and HCAZ scores.

Statistical analyses were conducted using SAS 9.4 statistical software (SAS Institute).

### 2.7 Ethical statement

The Women First trial was approved by the Colorado Multiple Institutional Review Board (#13‐2160); Comité de Ética Universidad Francisco Marroquin (CE‐FM/UFM 059‐17, Guatemala); JNMC Institutional Ethics Committee on Human Subjects Research and the Indian Council of Medical Research (KAHER/EC/2018‐19/D3017, India); Comité D'Ethique, Ecole De Sante Publique, University of Kinshasa (ES/CE/102B/14, Democratic Republic of the Congo); the Aga Khan University Ethical Review Committee (2753‐CHS‐ERC‐13, Pakistan); and RTI International (NC, USA). Mothers provided written informed consent for themselves and their children. The primary study protocol is available online at https://www.ncbi.nlm.nih.gov/pmc/articles/PMC4000057. The WF trial is registered at ClinicalTrials.gov with the following Identifier, NCT01883193. Details on ethics approval can be provided upon request.

## 3 RESULTS

### 3.1 Participant characteristics

Of the 2221 infants with valid birth measurements who were consented to the WF offspring follow‐up, 1488 were randomized for BSID‐III testing. Of those, 1386 offspring participants (95%) had at least one BSID‐III domain scored and were considered part of the analysis. Distribution of participants was balanced by arm, ranging from 30% to 33%, and by sex, with 49% male. The number of participants for each site was 271 in DRC, 391 in Pakistan, 356 in Guatemala and 368 in India, respectively (Supporting Information S1: Figure S1). Characteristics of the study population by intervention arm are shown in Table 1. Comparison of birth anthropometry for the infants in the BSID‐III subset to that of the offspring of the entire WF trial cohort according to intervention arm demonstrated no significant differences (Supporting Information S2: Table S1). Likewise, no differences in maternal age, body mass index, and education were detected between mothers of infants in the present subset and those of the entire trial cohort (Supporting Information S2: Table S1).

**Table 1:** Maternal characteristics and birth anthropometry for subset of offspring participants assessed with the Bayley Scales of Infant Development (BSID‐III) at 24 months of age by intervention arm.

Variable	Arm 1^a^	Arm 2	Arm 3
BSID‐III evaluations in offspring, *N* b	436	486	457
Maternal age, *N* (%)			
<20 years	87 (20.0)	101 (21.0)	91 (19.9)
20+ years	349 (80.0)	384 (79.0)	366 (80.1)
Maternal education, *N* (%)			
Secondary or more	146 (33.5)	155 (31.9)	127 (27.8)
Body mass index (BMI), kg/m^2^	21.3 ± 4.1	21.4 ± 4.1	21.5 ± 4.1
Height, cm	151.4 ± 6.5	151.2 ± 6.8	150.7 ± 6.8
Parity, *N* (%)			
0 (nulliparous)	104 (23.9)	85 (17.6)	83 (18.2)
≥1	331 (76.1)	400 (82.3)	374 (81.8)
Tally of indicators of higher SES^c^			
Low (0−2 present), *N* (%)	173 (39.7)	184 (37.9)	178 (38.9)
High (3−6 present), *N* (%)	263 (60.3)	302 (62.1)	279 (61.1)
Infant birth anthropometry, *N*	441	485	458
Length‐for‐age *z*‐score	−1.00 ± 1.11	−0.95 ± 1.06	−1.18 ± 1.12
Weight‐for‐age *z*‐score	−1.11 ± 1.02	−1.07 ± 0.96	−1.23 ± 0.95
LBW, *N* (%)	105 (24.0)	108 (22.3)	119 (26.0)

*Note*: Data presented as *N* (%) or mean ± SD. LBW, birthweight ≤2500 g.

^a^ Maternal participants of the Women First trial in Arm 1 started lipid‐based nutrition supplements ≥3 months before conception; Arm 2 started same supplement at ~11 weeks gestation; and Arm 3 (control) received no trial supplements. All supplements were discontinued at delivery. Comparisons among participants within intervention arms revealed no statistically significant differences for the variables shown (*p *> 0.05).

^b^ All infants in the 24‐month longitudinal subset with at least one reliable BSID‐III composite score and at least one 24‐month anthropometric outcome (length‐for‐age, weight‐for‐age, or head circumference‐for‐age *z*‐score).

^c^ The socioeconomic status (SES) tally provides the number of indicators available from the following list: Electricity, improved water source, sanitation, man‐made flooring, improved cooking fuels and household assets.

### 3.2 Aim 1: Neurodevelopmental outcomes by intervention arm

Treatment effects on BSID‐III scores by maternal intervention arm for individual sites according to developmental domain are shown in Table 2. For three of four sites, there was no effect of the intervention on child neurodevelopment outcomes. A site by arm interaction effect was identified for the DRC, with the intervention arm having a statistically significant effect for both cognitive and motor scores (*p* = 0.0025 and 0.0009, respectively) (Table 2). In this site only, the early pregnancy arm (Arm 2) was associated with lower adjusted mean differences than both the preconception (Arm 1) and the control arms (Arm 3). No significant interaction between infant sex and intervention arm was identified for any of the neurodevelopmental outcomes and thus this variable was excluded from the final model. Sensitivity analyses for the continuous variables indicated that with exclusion of DRC, there was no significant difference in any domain by intervention arm.

**Table 2:** Effect of intervention arm on composite development domain scores for the Bayley Scales of Infant Development (BSID‐III) according to site.^a^

	Arm 1 vs. Arm 2^b^		Arm 1 vs. Arm 3		Arm 2 vs. Arm 3	
Study site/ND domain	Adj mean diff (95% CI)	*p* Value	Adj mean diff (95% CI)	*p* Value	Adj mean diff (95% CI)	*p* Value
Guatemala						
Cognitive	−1.55 (−4.58, 1.48)	0.32	−1.00 (−4.07, 2.06)	0.52	0.54 (−2.35, 3.43)	0.71
Motor	−0.56 (−3.73, 2.62)	0.73	−1.45 (−4.68, 1.78)	0.38	−0.89 (−3.92, 2.13)	0.56
India						
Cognitive	1.24 (−1.66, 4.15)	0.40	−0.14 (−3.06, 2.78	0.92	−1.39 (−4.31, 1.54)	0.35
Motor	2.30 (−0.79, 5.38)	0.15	1.28 (1.82, 4.39)	0.42	−1.01 (−4.13, 2.10)	0.52
Pakistan						
Cognitive	0.68 (−2.18, 3.54)	0.64	0.30 (−2.60, 3.20)	0.84	−0.38 (−3.26, 2.50)	0.80
Motor	1.38 (−1.65, 4.40)	0.37	2.31 (−0.75, 5.38)	0.14	0.94 (−2.10, 3.98)	0.55
DRC						
Cognitive	**6.39 (2.82, 9.96)**	**<0.001**	−1.95 (−5.52, 1.61)	0.28	**−8.34 (−11.77, −4.91)**	**<0.001**
Motor	**8.14 (4.40, 11.87)**	**<0.001**	−0.53 (−4.29, 3.22)	0.78	**−8.67 (−12.26, −5.08)**	**<0.001**

Abbreviations: DRC, Democratic Republic of the Congo; ND, neurodevelopment.

^a^ Adjusted mean difference (Adj mean diff) and 95% confidence intervals (CIs) in domain specific BSID‐III scores by intervention arm according to site. For social‐emotional composite scores, the reference standard was Arm 3 (no interventional supplementation), thus no comparisons between Arms 1 and 2 were made for this domain. The final model included site, maternal education, maternal age, parity, socioeconomic status, infant sex and family care indicator subscales: Play activities (0–10), play materials (0–8) and household books (1–2, 3–5, ≥6) as covariates. Bolded values are those found significant for *p* < 0.05.

^b^ Maternal participants in Arm 1 started lipid‐based nutrition supplements ≥3 months before conception; Arm 2 started same supplement at ~11 weeks gestation; and Arm 3 (control) received no trial supplements.

Similar to results noted from the continuous data analysis, categorical assessments for risk of cognitive and motor delay for combined sites also indicated that Arm 2 was associated with a higher risk compared to the control Arm 1. In this analyses, maternal age, infant sex and play materials also had a significant effect on cognitive and motor neurodevelopment scores (Supporting Information S2: Table S2.

### 3.3 Aim 2: Predictors of neurodevelopmental outcomes

**Figure 1:**
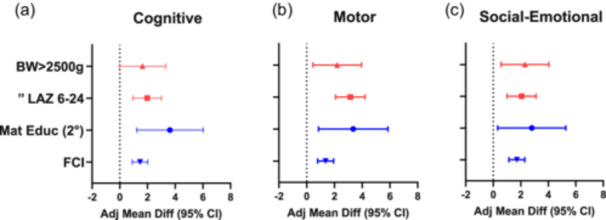
Anthropometric and sociodemographic covariates associated with neurodevelopmental outcomes in offspring of maternal participants in the Preconception Maternal Nutrition Intervention Trial (Women First). Legend: Birthweight (>2500 g), change in length‐for‐age *z*‐scores between 6 and 24 months of age (∆LAZ6‐24), maternal education (Mat Educ [2°]) and family care indicators (Play Materials) were significantly associated with Bayley Scales of Infant Development, 3rd edition (BSID‐III) cognitive (a), motor (b), and social‐emotional (c) domains. Using linear (robust Poisson) regression for continuous outcomes, *p* < 0.05, and 95% confidence intervals (CI), associations with neurodevelopment scores for combined sites at 24 months of age were identified. Values shown are adjusted mean differences (adj mean diff ± 95% CI) across all subscales. The final model included the following covariates: intervention arm, site, maternal secondary education (i.e., secondary maternal education was compared to no formal schooling to produce the adj mean diff), maternal age, parity, socioeconomic status, infant sex, family care indicator subscales (play activities [0–10], variety of play materials [0–8], sources of play materials [0–3] and household books [1–2, 3–5, ≥6]), low‐birthweight, ∆LAZ6‐24 and anthropometric *z*‐scores.

Four covariates were significantly and consistently associated with neurodevelopmental outcomes on all subscales, including birthweight >2500 g (trend for cognitive domain), change in LAZ from 6 to 24 months (ΔLAZ_6__–__24_), maternal secondary education, and FCI play materials (Figure 1). Neither LAZ nor WAZ at 6 months, nor maternal primary education were consistently associated with BSID‐III subscale scores across all domains (Supporting Information S2: Table S3). The presence of stunting (LAZ <−2) and/or wasting (WLZ <−2) at 6 months was not significantly associated with any of the neurodevelopment domain scores at 24 months, though not being wasted was marginally associated (*p* = 0.05) with motor development scores at 24 months.

### 3.4 Aim 3: Relationship of growth to neurodevelopmental outcomes

**Figure 2:**
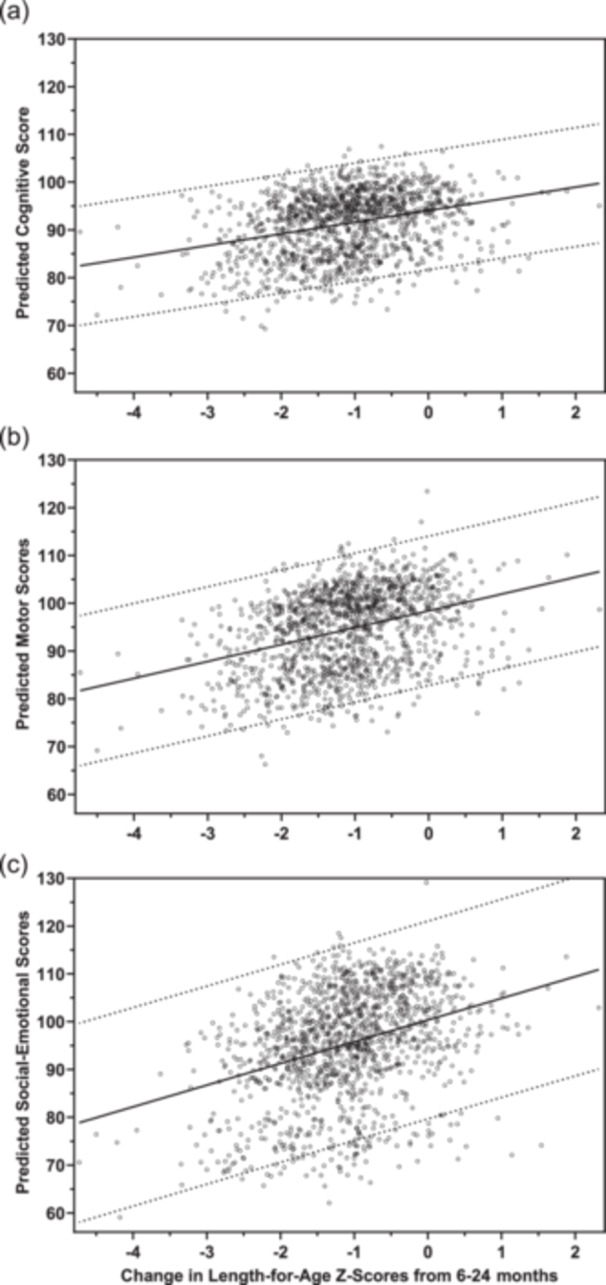
Scatter plots of adjusted predicted Bayley Scales of Infant Development, 3rd edition (BSID‐III) cognitive, motor and social‐emotional scores versus change in length‐for‐age *z*‐scores from 6 to 24 months (∆LAZ_6–24_). (a) Adjusted predicted cognitive scores versus ∆LAZ_6–24_; (b) Adjusted predicted motor scores versus ∆LAZ_6–24_; (c) Adjusted predicted social‐emotional scores versus ∆LAZ_6–24_. Models adjusted for intervention arm, site, cluster, interaction between site and cluster, maternal education, maternal age, parity, socioeconomic status (SES), infant sex and family care indicator subscales: play activities (0–10), play materials (0–8), and household books (1–2, 3–5, ≥6); change in length‐for‐age *z*‐scores (∆LAZ_6–24_), weight‐for‐age *z*‐score, (∆WAZ_6‐24_) and head circumference‐for‐age *z*‐score (∆HCAZ_6–24_) from 6 months to 24 months of age; and LAZ <−2, weight‐for‐length *z*‐score (WLZ) <−2.

Of the three anthropometric indices, only the change in LAZ score from 6 to 24 months was directly associated with all three of the BSID‐III subscales across all sites (Figure 2). For every 1‐point change in ΔLAZ_6__–__24_, the adjusted mean differences in the cognitive, motor and social‐emotional composite scores were 1.98 (95% CI 0.96, 3.01); 3.15 (95% CI 2.08, 4.21), and 2.06 (95% CI 1.00, 3.12), respectively (*p* < 0.0002 for all scales).

## 4 DISCUSSION

This study examined the potential for enduring effects of a maternal nutrition intervention initiated before conception, early in pregnancy, or not at all on neurodevelopment of offspring assessed at 2 years. Contrary to our hypothesis, the results did not support a benefit of the nutrition intervention on any of the developmental outcomes, including cognitive, motor and social‐emotional domains. For our second objective, to determine anthropometric and sociodemographic predictors of neurodevelopmental scores, we identified four covariates that were associated with scores in all three domains: maternal education; linear growth between 6 and 24 months (indicated by change in LAZ score); birthweight above 2500 grams; and extent of access to household play materials. For the third objective, examination of associations between anthropometric indicators and neurodevelopment subscales, only the change in LAZ from 6 to 24 months was found to have a significant positive association with scores for all developmental domains. These findings point to multi‐dimensional pre‐ and post‐natal factors as important for early childhood development.

The absence of a detectable effect of the maternal nutrition intervention may indicate the limits of the impact of improved maternal nutrition early in pregnancy on the rapid, nutrition sensitive development that occurs during the last trimester and post‐natally (Thompson & Nelson, 2001). For example, myelination and hippocampal growth sharply increase in the third trimester and actively continue through the first approximately 24 post‐natal months, and prefrontal cortex growth is sharpest in the first 6 post‐natal months (Cusick & Georgieff, 2016). First trimester events, such as neurogenesis and neural migration are susceptible to micronutrient deficiencies, for example both iodine (Cusick & Georgieff, 2016) and zinc (Brion et al., 2021). Comparisons of maternal status for both of these micronutrients demonstrated small advantages for the preconception arm at 12 weeks' gestation (Young et al., 2021). Yet, differences in mean micronutrient status were small and varied by site, and any beneficial impact of the intervention for these and other micronutrients during early gestation may have been overwhelmed by the impoverished conditions in all sites where the study was conducted. All sites had very high rates of post‐natal growth faltering and stunting (Krebs et al., 2022), poor quality of complementary feeding (Long et al., 2023) and low levels of maternal education (Hambidge et al., 2019). Our findings differ slightly from the 2‐year follow‐up of another preconception maternal supplementation trial in which scores of the fine motor scale (only) were higher in the offspring of mothers who received an iron‐folate supplement compared to those who received either folate alone or a multiple micronutrient supplement (Nguyen et al., 2017). The current results are, however, consistent with a recent systematic review of SQ‐LNS trials that concluded maternal supplementation had minimal beneficial impact on developmental outcomes (Prado et al., 2021).

The results from the DRC were an exception to the lack of difference observed among the three intervention arms. For this site, the prenatal supplementation arm (Arm 2) was associated with significantly worse developmental outcomes compared to both the preconception and control arms. We are unable to explain this anomalous effect based on any detectable data quality issues, biologic plausibility, or published literature (Nguyen et al., 2017; Prado et al., 2016; Prado, Abbeddou, Adu‐Afarwuah, et al., 2017; Prado et al., 2021). Thus, while no objective indication justified exclusion of DRC data and the data from the site were retained, we conclude that the study's results are most consistent with an absence of an effect of the maternal intervention on neurodevelopmental outcomes. This conclusion is consistent with outcomes of the INTER‐NDA combined site analysis that evaluated the other one‐third of the eligible offspring of WF participants (Fernandes et al., 2023). In that assessment, no differences in cognitive, language, gross motor, fine motor or behaviour scores at 24 months were identified by maternal intervention arm.

Of the multiple covariates considered for predictive value for neurodevelopmental outcomes, secondary maternal education at enrolment was the strongest predictor of a higher adjusted mean difference in each of the domains examined, with adjusted mean differences greater than three points for both cognitive and motor composite scores. This finding has not been a consistent one in multiple studies of predictors of early childhood development (Nguyen et al., 2017; Prado et al., 2016) and may reflect the very limited education levels of the WF participants (mean of 4–5 years), with only ~1/3 of women having secondary education. Notably, maternal education was also associated with multiple early childhood development outcomes in these diverse setting using the INTER‐NDA assessment tool (Fernandes et al., 2023). A recent systematic review of SQ‐LNS interventions also posited that post‐natal supplementation may yield greater effects for children whose mothers had lower educational attainment (Prado et al., 2021).

The next most potent predictor of neurodevelopment was linear growth from 6 to 24 months, which was associated with higher adjusted mean differences of 2–3 units for each domain in our study. Similarly, the LAZ at 24 months was significantly associated with subscores for the children randomized to the INTER‐NDA study (Fernandes et al., 2023). Linear growth from early infancy into the second year of life has previously been associated with early child development, in both intervention (Prado et al., 2016; Prado, Abbeddou, Adu‐Afarwuah, et al., 2017; Prado et al., 2019; Prado et al., 2021) and observational studies (Donowitz et al., 2018; Scharf et al., 2018; Sudfeld et al., 2015). Settings with high rates of stunting (>35%) have demonstrated greater responses to post‐natal SQ‐LNS interventions (Prado et al., 2021). Stunting rates at 24 months averaged over 60% for the sites in the WF trial (Krebs et al., 2022), and thus improved post‐natal nutrition or supplementation, which was not undertaken in this study, may potentially have benefitted the early development of the children in our study (Long et al., 2023).

The other biomedical indicator with significant predictive effect and association in developmental domains was birthweight greater than 2500 g, that is, not being low‐birthweight. Since low‐birthweight can reflect both fetal growth restriction and prematurity, it is possible that this predictor was influenced by the latter, since we did not adjust for gestational age in our developmental evaluations and analyses. Birthweight, unadjusted for gestational age, was also found to be associated with 24‐month cognitive scores in the multisite MAL‐ED study (Scharf et al., 2018). Moreover, growth faltering at any time during the first 1000 days, including pregnancy, has been associated with poor early child development outcomes (Prado et al., 2016).

Finally, access to household play materials, assessed as a component of the FCI, was a consistent significant independent predictor of scores in all the developmental domains we evaluated, with the highest adjusted mean difference for the social‐emotional subscale. As demonstrated by post‐natal nutrition intervention trials, the association of linear growth and early child development is associated with home and environmental factors, including access to stimulating play materials (Prado et al., 2016). The importance of this family environment indicator is likely its reflection of parental–child interactions. Various pre‐ and post‐natal maternal and infant intervention studies have found limited influence of nutrient supplementation on neurodevelopment but have shown stronger and perhaps compensatory effects of emotionally supportive, developmentally stimulating home environments that offer age‐appropriate opportunities for play and exploration (Black et al., 2017, 2021; Trude et al., 2021). A systematic review and meta‐analysis found interventions promoting such ‘nurturing care’ and learning opportunities had effects on cognitive, motor and language scores that were four to five times larger than those resulting from nutritional supplementation alone (Prado et al., 2019).

As expected on the basis of extant literature (Prado et al., 2016; Scharf et al., 2018; Sudfeld et al., 2015), we found a robust positive relationship between linear growth over the 18 months before testing and each of the domains of neurodevelopment. The strength of associations was considerably greater compared to another multisite analysis that had more variable stunting burdens (Scharf et al., 2018). No indicators of ponderal growth or head circumference were associated, although the former was highly correlated with LAZ.

The associations we have identified between linear growth and neurodevelopment do not infer causality. Rather, our observations are consistent with the conclusion that factors driving somatic growth and development are shared but distinct. Anabolic stimulation via both nutritional and hormonal processes (e.g., growth hormone and insulin‐like growth factor‐1) drive growth if it is not constrained by pathophysiologic factors, and these may have corollary effects on neurodevelopmental pathways (Gunnell et al., 2005). In the present study, linear growth from 6 to 24 months was also a strong predictor of neurodevelopmental outcomes. Moreover, as previously reported, birth length was positively associated with the maternal intervention and with post‐natal linear growth trajectory over the first 2 years of life (Krebs et al., 2022).

Strengths of this study include the individual participant randomization of the original preconception trial (Hambidge et al., 2019) and the randomized selection of offspring of the WF participants for the neurodevelopmental testing; prospective longitudinal design, including multiple periodic anthropometric measurements from birth to the time of the developmental testing; high completion rate for the randomized participants; and use of a standardized training and monitoring protocol for administration of the BSID‐III over four diverse LMIC sites. Limitations are also noted, however, including unmeasured potentially important confounding factors such as maternal and/or child anemia and other biomarkers of nutritional status; the use of WHO anthropometric standard *z*‐scores, which lack adjustment for gestational age; and lack of data on childhood infectious morbidity and enteric disease (Mal‐Ed Network Investigators, 2018).

## 5 CONCLUSION

In conclusion, the constellation of findings from the present analysis reinforces the premise that multiple factors drive improvements in early child development across multiple domains. Overall, in this randomized controlled trial conducted in settings with notably high rates of stunting, the provision of preconception and early prenatal maternal nutrition supplementation resulted in improved fetal and post‐natal linear growth, and in turn, these benefits were significantly associated with neurodevelopment outcomes across all domains evaluated. This suggests potential for some enduring downstream benefits of the maternal intervention for early child neurodevelopment. The associations of maternal education and child access to play materials highlight the importance of these critical components of nurturing care for early child development, that is, opportunities for learning in the home and social environment and the influence of the parental caregiver. These components, combined with healthy birthweight and optimized post‐natal linear growth, positively impact early child development in low‐ and middle‐income countries. Thus, multi‐pronged programmatic efforts, as represented in the nurturing care framework, are likely to yield more potent and persistent impacts on children's developmental capacity than more narrowly focused interventions.

## AUTHOR CONTRIBUTIONS

Nancy F. Krebs and Michael Hambidge conceived and designed the study; Nancy F. Krebs, Michael Hambidge and Jamie E. Westcott wrote the final protocol in collaboration with all members of the trial group (Fred Biasini, Dhuly Chowdhury, Ana Garcés, Lester Figueroa, Antoinette Tshefu, Adrien Lokangaka, Melissa Bauserman, Sarah Saleem, Sumera A. Ali, Robert L. Goldenberg, Shivaprasad S. Goudar, Sangappa M. Dhaded, Richard J. Derman, Jennifer F. Kemp, Kristen Stolka, Marion Koso‐Thomas, Abhik Das and members of the Women First Preconception Nutrition Trial Group listed below); Fred Biasini provided expert training to neurodevelopment assessors in each site; Ana Garcés, Lester Figueroa, Antoinette Tshefu, Adrien Lokangaka, Sarah Saleem, Sumera A. Ali, Omrana Pasha, Shivaprasad S. Goudar and Sangappa M. Dhaded coordinated implementation of the study at the country level; Nancy F. Krebs, Michael Hambidge, Melissa Bauserman, Carl L. Bose, Robert L. Goldenberg and Richard J. Derman provided overall supervision of study conduct; Aura Arevalo, Gelen Gomez, Marta Lidia Aguilar, Zahid Abbasi, Sumera Aziz Ali, Sumaira Fatima, Deepa Metgud, Spurthi Mastiholi, Robert Kpado, Robert Kpado, Matthieu Gbozo, Philippe Zolia, Papy Fakadanga and Joel Eay performed developmental assessments; Stephanie Waldrop and Nancy F. Krebs drafted the manuscript with critical input from all authors for subsequent revisions; Jennifer F. Kemp supported data base management and statistical analyses; Dhuly Chowdhury, Abhik Das, Amaanti Sridhar, Elizabeth M. McClure and Vanessa R. Thorsten provided statistical analyses. All authors read and approved the final version of the manuscript. Portions of these data were presented in abstract form at the annual meeting of the American Society for Nutrition (‘Nutrition 2022’) in June 2022, Baltimore, MD. (Krebs et al., 2022).

## THE WOMEN FIRST MATERNAL PRECONCEPTION NUTRITION TRIAL STUDY GROUP

The Women First Maternal Preconception Nutrition Trial Study Group consists of the following additional members: Carl L. Bose MD (University of North Carolina, Chapel Hill, NC, USA); Neurodevelopment Assessors: Aura Arevalo, Gelen Gomez PhD, and Marta Lidia Aguilar PhD (INCAP, Guatemala); Zahid Abbasi PhD, and Sumaira Fatima PhD (Aga Khan University, Karachi, Pakistan); Deepa Metgud PhD and Spurthi Mastiholi PhD (KLE Academy of Higher Education and Research (Deemed to be University) Jawaharlal Nehru Medical College, Belagavi, India); Robert Kpado BS, Croco Gbenge BS, Matthieu Gbozo BS, Philippe Zolia BS, Papy Fakadanga BS, and Joel Eay BS (Kinshasa School of Public Health, Kinshasa, Democratic Republic of the Congo); Elizabeth McClure PhD, Vanessa R. Thorsten MPH, Amaanti Sridhar MS, and Kristen Stolka MPH (RTI International, Durham, NC, USA); Sarah Borengasser PhD (University of Colorado School of Medicine, Aurora, CO, USA); Omrana Pasha MD (Aga Khan University, Karachi, Pakistan); Manjunath Somannavar MD (KLE Academy of Higher Education and Research (Deemed to be University) Jawaharlal Nehru Medical College, Belagavi, India); Marion Koso‐Thomas (National Institute of Child Health and Human Development/NIH, Bethesda, MD, USA).

## CONFLICT OF INTEREST STATEMENT

The authors declare no conflict of interest.

## Supporting information

Supporting information.

Supporting information.

## Data Availability

De‐identified study data will be available through the National Institute of Child Health and Human Development Data and Specimen Hub at https://dash.nichd.nih.gov.
