# Supplementary material for: Anthropometric and sociodemographic variables, but not preconception or prenatal maternal nutrition supplementation, predict neurodevelopment in offspring of the ‘Women First’ trial
Source: Matern Child Nutr. 2024 Jul 23;20(4):e13703. doi: 10.1111/mcn.13703 (PMC11574664; doi:10.1111/mcn.13703)
Supplement: Supplementary file 1 — Supporting information. [file MCN-20-e13703-s001.pdf]

## Appendix 1

**Supplementary Figure S1.** CONSORT diagram showing screening, consent, randomization, and longitudinal analysis subset [a] by intervention arm

**Supplementary Figure 1**

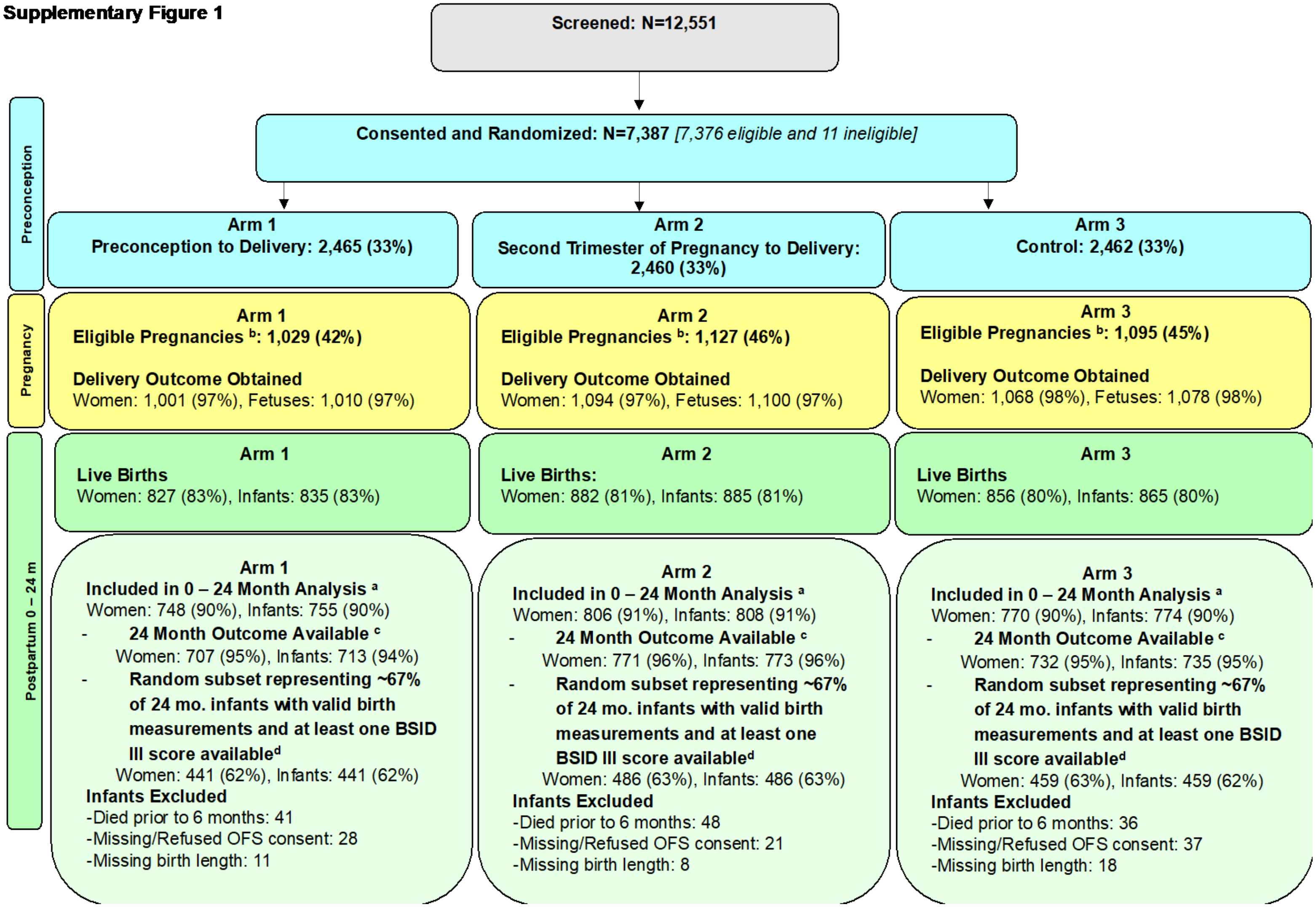

[a] After excluding extreme invalid measurements as determined by expert manual review and accounting for biologically implausible z-scores based on WHO standards [e], the 24-month longitudinal analysis subset includes all live born infants with birth length measurements measured by 7 days (168 hours) of age on portable length boards and consented to the offspring follow-up study.

[b] Excludes women who became pregnant less than 3 months into the study. The women who had eligible pregnancies may have had delivery data obtained or they may have exited the study prior to delivery.

[c] At least one of the following anthropometric outcomes must be available after excluding extreme invalid measurements as determined by expert manual review and accounting for biologically implausible z-scores based on WHO standards[e]: length, weight, head-circumference, weight-for-length measurements.

[d] Those infants with at least one BSID III score available and thus included and assessed in the sub cohort that consented to the Women First Neurodevelopment Follow-up Study

[e] Extreme invalid measurements as determined by expert manual review were excluded from the longitudinal analysis. All length-for-age, weight-for-age, weight-for-length, and head-circumference-for-age z-scores (LAZ, WAZ, WLZ, and HCAZ respectively) are calculated using the expanded tables of the Child Growth Standards published by the WHO that provide z-scores by sex and age in days at time of measurement. All WHO standards are based on term infants. LAZ, WAZ, WLZ, and HCAZ are within the biologically plausible range according to WHO standards ($-6 \leq LAZ \leq 6$, $-6 \leq WAZ \leq 6$, $-5 \leq WLZ \leq 5$, $-5 \leq HCAZ \leq 5$). If an infant is found to have a biologically implausible LAZ or WAZ according to WHO standards at a visit, all growth outcomes at the visit are set to missing. If an infant is found to have a biologically implausible WLZ or HCAZ according to WHO standards at a visit, only the corresponding measurement and z-score at the visit are set to missing. WLZ could not be obtained for infants with a length of less than 45.0 cm at any visit due to limitations in the WHO standards and were set to missing for that visit. (http://www.who.int/tools/child-growth-standards).
