# Supplementary material for: Anthropometric and sociodemographic variables, but not preconception or prenatal maternal nutrition supplementation, predict neurodevelopment in offspring of the ‘Women First’ trial
Source: Matern Child Nutr. 2024 Jul 23;20(4):e13703. doi: 10.1111/mcn.13703 (PMC11574664; doi:10.1111/mcn.13703)
Supplement: Supplementary file 2 — Supporting information. [file MCN-20-e13703-s002.pdf]

Appendix 2

Supplementary Table S1. Comparison of baseline and anthropometric characteristics between the neurodevelopment BSID-III study offspring cohort and the Women's First (WF) primary study cohort according to intervention arm.†

	**WF Arm 1**	**BSID-III Arm 1**	**P-value‡**
**N**	**2337**	**441**	
**LAZ§**	-1.05 ± 1.22	-1.00 ± 1.11	0.970
**WAZ§**	-1.13 ± 1.06	-1.11 ± 1.02	0.989
**% LBW§**	563 (24.1%)	105 (24.0%)	0.952
	**WF Arm 2**	**BSID-III Arm 2**	**P-value$^{\delta}$**
**N**	**2337**	**485**	
**LAZ**	-1.02 ± 1.11	-0.95 ± 1.06	0.964
**WAZ**	-1.12 ± 1.01	-1.07 ± 0.96	0.971
**% LBW**	537 (23.0%)	108 (22.3%)	0.767
	**WF Arm 3**	**BSID-III Arm 3**	**P-value$^{\delta}$**
**N**	**2337**	**458**	
**LAZ**	-1.22 ± 1.14	-1.18 ± 1.12	0.980
**WAZ**	-1.25 ± 1.01	-1.23 ± 0.95	0.989
**% LBW**	635 (27.2%)	119 (26.0%)	0.645

Values presented as mean ± SD or N (%)

[‡]P-value level of significance was set at 0.05. Continuous variables were assessed with the Kruskal-Wallis or Welch's ANOVA tests and categorical variables with the Fischer's Exact test.

[§]LAZ, length-for-age z-score; WAZ, weight-for-age z-score; %LBW, percent of participants with low birth weight, defined as a weight of $\leq$ 2500g at birth.

Supplementary Table S2. Effect of intervention arm, sociodemographic indicators, and Family Care Indicators (FCI) on risk of delayed development for composite cognitive and motor score for all sites†

	**Cognitive**<br><br>**Odds Ratio (95% CI)**	**P value**	**Motor**<br><br>**Odds Ratio (95% CI)**	**P value**
Arm 1 vs Arm 2‡	**0.49 (0.28, 0.86)**	**0.013**	**0.46 (0.27, 0.81)**	**0.007**
Arm 1 vs Arm 3	1.01 (0.55, 1.85)	0.970	0.84 (0.47, 1.53)	0.577
Arm 2 vs Arm 3	**2.05 (1.19, 3.53)**	**0.010**	**1.82 (1.08, 3.09)**	**0.025**
Maternal education§	0.48 (0.19, 1.19)	0.113	0.42 (0.17, 1.05)	0.063
Maternal age (>20 y)	**2.74 (1.28, 5.89)**	**0.010**	1.84 (0.89, 3.81)	0.098
Parity (ref nulliparous)	0.77 (0.40, 1.49)	0.432	1.06 (0.55, 2.07)	0.859
Infant sex (ref female)	**0.57 (0.36, 0.90)**	**0.017**	**0.50 (0.32, 0.79)**	**0.003**
Play materials (FCI)	**0.74 (0.62, 0.87)**	**0.0004**	**0.75 (0.64, 0.89)**	**0.001**
Play activities (FCI)	0.99 (0.85, 1.14)	0.835	1.04 (0.90, 1.19)	0.616

†Categorical analysis of Bayley Scales of Infant Development, 3rd edition (BSID-III) cognitive and motor domain scores using cutoff levels of <75, indicating risk of delayed development for all sites. Model included the following covariates: intervention arm, site, maternal education, maternal age, parity, infant sex, and family care indicator subscales (i.e., play activities (0-10) and play materials (0-8). Bolded values are those found significant for $p < 0.05$

‡Maternal participants in Arm 1 started lipid-based nutrition supplements ≥ 3 months prior to conception; Arm 2 started same supplement at ~11 weeks gestation; and Arm 3 (control) received no trial supplements.

§Maternal secondary education is compared to no formal schooling for both cognitive and motor composite score outcomes.

Supplementary Table S3. Anthropometric, sociodemographic and Family Care Indicator (FCI) predictors of BSID-III[†] neurodevelopmental composite domain scores for all sites[‡]

Predictor Variables	Cognitive domain		Motor domain		Social/emotional domain	
	Adj mean diff (95%CI)	P value	Adj mean diff (95%CI)	P value	Adj mean diff (95%CI)	P value
**Anthropometric predictors**						
$LAZ_{6\ mo}$[§]	-0.67 (-1.55, 0.21)	0.135	-0.35 (-1.27, 0.57)	0.455	**-0.99 (-1.90, -0.09)**	**0.031**
$WAZ_{6\ mo}$[§¶]	**1.07 (0.20, 1.93)**	**0.016**	**1.47 (0.57, 2.37**	**0.001**	0.88 (-0.01, 1.77)	0.054
$HCAZ_{6\ mo}$[§]	-0.19 (-0.96, 0.58)	0.63	-0.23 (-1.03, 0.58)	0.582	0.12 (-0.68, 0.92)	0.777
**Sociodemographic predictors**						
Infant male sex	0.82 (-0.45, 2.09)	0.204	0.84 (-0.49, 2.16)	0.216	**-1.91 (-3.21, -0.61)**	**0.004**
Maternal primary education	**2.64 (0.53, 4.74)**	**0.014**	1.53 (-0.68, 3.74)	0.174	-0.38 (-2.55, 1.80)	0.735
Socioeconomic status	-0.12 (-0.75, 0.50)	0.701	0.19 (-0.47, 0.84)	0.58	-0.24 (-0.88, 0.40)	0.463
**Family Care Indicators**						
Play activities	0.27 (-0.12, 0.67)	0.176	0.12 (-0.29, 0.53)	0.564	**0.53 (0.13, 0.93)**	**<0.01**

[†]BSID-III, Bayley Scales of Infant Development, 3rd edition

[‡]Values are adjusted mean difference (adj mean diff) with 95% CIs. Bolded values are those significant for $p < 0.05$. Model includes analysis for site, intervention arm, maternal education, maternal age, parity, socioeconomic status, infant sex, family care indicators subscales (i.e., play activities, play materials, number of household books (i.e.,1 to 2, 3 to 5, ≥6).

[§]LAZ_{6mo}, length-for-age z-score at age 6 months; $HCAZ_{6mo}$, head circumference-for-age z-score at age 6 months; WAZ_{6mo}, weight-for-age z-score at age 6 months.

[¶]WAZ_{6mo} was excluded from the final model as it was highly correlated with LAZ_{6mo} and $HCAZ_{6mo}$
